# Potent microtubule depolymerizing activity of a mitotic Kif18b-MCAK-EB network

**DOI:** 10.1242/jcs.260144

**Published:** 2022-05-31

**Authors:** Toni McHugh, Julie P.I. Welburn

**Affiliations:** 1 Wellcome Trust Centre for Cell Biology, School of Biological Sciences, University of Edinburgh, Edinburgh EH9 3BF, Scotland, UK

## Abstract

The precise regulation of microtubule length during mitosis is essential to assemble and position the mitotic spindle and segregate chromosomes. The kinesin-13 Kif2C/MCAK acts as a potent microtubule depolymerase diffusing short distances on microtubules, while the kinesin-8 Kif18b is a processive motor with weak depolymerase activity. However, the individual activities of these factors cannot explain the dramatic increase in microtubule dynamics in mitosis. Using *in vitro* reconstitution and single molecule imaging, we demonstrate that Kif18b, MCAK and the plus-end tracking protein EB3 act in an integrated manner to potently promote microtubule depolymerization at very low concentration. We find that Kif18b can transport EB3 and MCAK and promotes their accumulation to microtubule plus ends through multivalent weak interactions. Together, our work defines the mechanistic basis for a cooperative Kif18b-EB-MCAK network at microtubule plus ends, that acts to efficiently shorten and regulate microtubules in mitosis, essential for correct chromosome segregation.

## Introduction

Throughout eukaryotes, length control of microtubule polymers is essential. Microtubule length is controlled through the regulation of microtubule dynamics by motors and microtubule-associated proteins (Mitchison and Kirschner, 1984). At the onset of mitosis, microtubule catastrophe frequency increases dramatically, leading to disassembly of the interphase microtubule network (Belmont et al., 1990; Piehl and Cassimeris, 2003). These dynamic properties of microtubules are essential to enable the dynamic assembly and remodeling of the mitotic spindle throughout mitosis and promote chromosome alignment and segregation. In most eukaryotes, ranging from yeast to parasites and humans, the increase in catastrophe frequency is driven by upregulation of microtubule-depolymerizing kinesin motors. These motors shorten microtubules, by promoting their catastrophe (reviewed in Walczak et al., 2013). There are two major families of depolymerizing kinesins: Kinesin-8 and Kinesin-13. Generally, absence of these motors is associated with defects in cell division (Gupta et al., 2006; Maney et al., 1998; Walczak et al., 1996). Yeast have Kinesin-8 motors only: Kip3 and KlpB from budding yeast and *A.nidulans* respectively, both essential for spindle positioning (Cottingham and Hoyt, 1997; Miller et al., 1998; Rischitor et al., 2004; Walczak et al., 1996). Metazoan species such as *D.melanogaster* have both Kinesin-8 (Klp67) and Kinesin-13 (Klp10, Klp59D) microtubule depolymerases with key roles in spindle assembly and positioning (Savoian and Glover, 2010; Goshima et al., 2005).

In mammalian cells, the Kinesin-13 family is made of 3 members: Kif2a, Kif2b and Kif2c/MCAK. Kif2a is present at centrosomes and weakly at kinetochores (Cameron et al., 2006), and Kif2b is absent from most cells and appears as a weak microtubule depolymerase in human cells (Tanenbaum et al., 2009; Welburn and Cheeseman, 2012). Kif2c/MCAK is the most potent and best-characterized microtubule-depolymerizing kinesin (Ems-McClung and Walczak, 2010; Friel and Welburn, 2018; Walczak et al., 2013). MCAK is strongly localized to centromeres in mitosis (Walczak et al., 1996; Wordeman et al., 1999). The endogenous localization of MCAK was reported cytoplasmic in interphase and at microtubule ends in mitosis (Moore et al., 2005; Tanenbaum et al., 2011; Wordeman et al., 1999). Most studies use transiently transfected MCAK tagged with a genetically encoded fluorophore to image MCAK plus end targeting to microtubules (Moore et al., 2005). *In vitro*, rather than walking on microtubules, MCAK diffuses short distances on the microtubule lattice (Helenius et al., 2006), raising the question of how MCAK reaches the ends of crowded microtubules in the dense cytoplasm in cells. *In vitro*, this atypical motor associates with microtubule end-binding (EB) proteins to enhance its localization to microtubule plus ends, utilizing an SxIP-like motif, while in cells disruption of this motif prevents the ability of GFP-MCAK to accumulate at microtubule ends (Honnappa et al., 2009; Montenegro Gouveia et al., 2010; Moore et al., 2005). EB3-dependent accumulation of MCAK at microtubule plus ends increases microtubule catastrophes (Montenegro Gouveia et al., 2010). However, the affinity of EB proteins for MCAK is low, around 10μM (Buey et al., 2012).

The human Kinesin-8 family has three members that regulate microtubule length: Kif19 at cilia, Kif18a, on kinetochore-fibres and Kif18b on astral microtubules (reviewed in Shrestha et al., 2018). The Kinesin-8 motor Kif18b shortens astral microtubules in mitosis when it is released into the cytoplasm from the nucleus (Lee et al., 2010; McHugh et al., 2018; Tanenbaum et al., 2011; Walczak et al., 2016). It requires both EB proteins and its C-terminal microtubule-binding tail to accumulate at microtubule ends and depolymerize them (McHugh et al., 2018; Stout et al., 2011; Tanenbaum et al., 2011). Absence of Kif18b or MCAK leads to aberrant microtubule growth, defects in spindle assembly and positioning, and lagging chromosomes (Domnitz et al., 2012; Huang et al., 2007; McHugh et al., 2018; Rankin and Wordeman, 2010). While Kif18b shortens microtubule length through its depolymerase activity, this activity *in vitro* remains modest compared to Kinesin-13 motors and does not explain the strong depolymerization associated with Kif18b function *in vivo*. Here using purified proteins, we show that Kif18b uses its motile properties to move MCAK and EB3 and promote their accumulation to microtubule plus ends. We also observe that MCAK and EB3 influence the motor properties of Kif18b. We reconstitute microtubule dynamics in the presence of Kif18b, EB3 and MCAK allowing us to dissect contributions of each factor to the activity of the network on microtubules and at microtubule plus ends. When all three components of the network are present at microtubule plus ends, we observe a large increase in catastrophe frequency which results in a significant decrease in microtubule length. Overall, it is the collective effect of the Kif18b, EB3 and MCAK on microtubules that enables robust change in microtubule dynamics and reduces microtubule length in mitosis.

## Results

### Kif18 is required for MCAK microtubule plus end localization in cells

**Figure 1:**
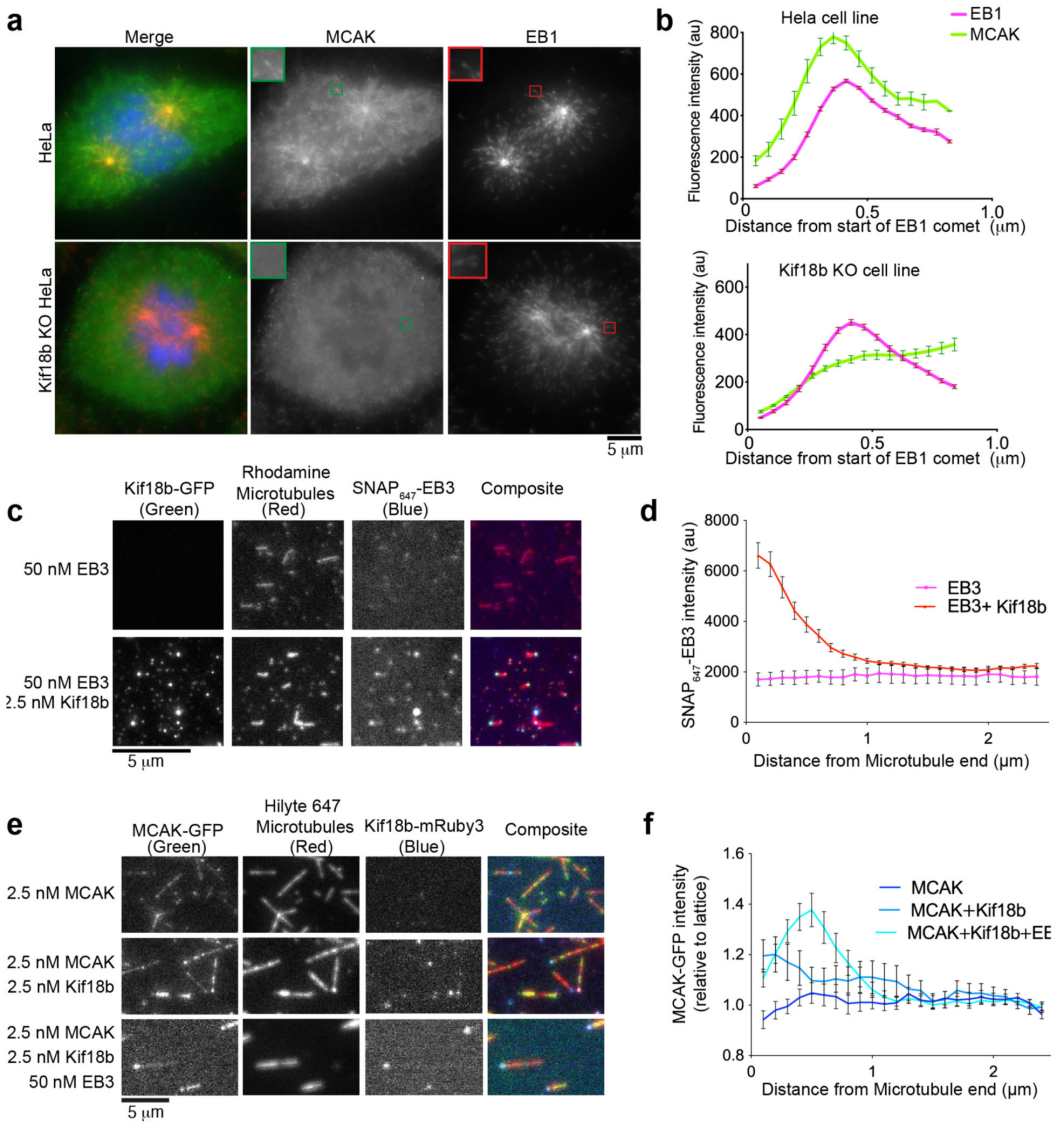
Kif18b increases the localization of MCAK at microtubule plus ends. a) Representative immunofluorescence images of MCAK (green) and EB1 (red) localization in HeLa and Kif18b Knockout Hela cells. Scale Bar 5 μm. b) Quantification of EB1 and MCAK mean fluorescence intensity at astral microtubule plus ends (n=30 and 27, respectively), repeated 3 times from independent experiments. Mean and standard error (S.E.) are represented. c & d) Kif18b binds to EB3 on GMPCPP microtubules. c) Representative images of Kif18b-GFP and SNAP_647_-EB3 on GMPCPP stabilized rhodamine labeled microtubules. Scale Bar, 5 μm. d) Quantification of EB3 fluorescence intensity at the microtubule plus end in the presence (red, n=41) or absence (pink, n=44) of 12.5 nM Kif18b-GFP (mean and standard deviation: S.D). e & f) Kif18b and EB3 increase the plus end localization of MCAK. e) Representative images of MCAK-GFP (green) localization on taxol and GMPCPP stabilized microtubules. Scale Bar 5 μm. f) Quantification of 12.5 nM MCAK-GFP fluorescence intensity (mean and S.E.) at microtubule plus ends relative to the lattice, alone (dark blue, n=76), in the presence of 12.5 nM Kif18b-mRuby3 (blue, n=90), or 12.5 nM Kif18b-mRuby3 and 50 nM SNAP_647_-EB3 (cyan, n=116). Kruskal Wallis test at 0.2 μm, MCAK versus Kif18b + MCAK, * P=0.0186, MCAK versus Kif18b + MCAK + EB3, **** P<0.0001.

First we investigated the localization of endogenous MCAK at microtubule plus ends during mitosis. In HeLa cells, we observed co-localization of MCAK with endogenous EB1 that marks the plus ends of astral microtubules. We then examined the localization of MCAK in a Kif18b knockout cell line that we had previously generated using Cas9-induced engineering, whose altered properties could be reversed by re-expressing Kif18b (McHugh et al., 2018). In cells lacking Kif18b (Fig S1a), MCAK was significantly reduced at microtubule plus ends (Fig 1a, b). These data, alongside previous work which showed MCAK microtubule plus end reduction after siRNA depletion of Kif18b (Tanenbaum et al., 2011), indicate that the localization of MCAK to microtubule plus ends is compromised in mitosis when Kif18b is absent both long and short-term (Fig 1a, b).

### Kif18b localizes MCAK and EB3 to stable microtubule plus ends

While MCAK requires Kif18b for plus end localization, the molecular mechanism underlying this dependency is not known, despite a weak interaction reported (Tanenbaum et al., 2011). The high-resolution molecular structure of dimeric MCAK is unknown. MCAK has a complex conformation in solution stabilized through intramolecular interactions, which could reduce the interaction with Kif18b (Ems-McClung et al., 2013; Maney et al., 2001; McHugh et al., 2019; Zong et al., 2016). We therefore examined the Alphafold2-predicted model structure for monomeric MCAK, which revealed a large part of the motor outside the catalytic domain is unstructured (Fig S1b) (Jumper et al., 2021). This model correctly predicts the far C terminus forms a small helix and interacts with the motor through conserved residues, which was previously shown in both Xenopus and human MCAK (Talapatra et al., 2015; Zong et al., 2016). We then used Alphafold2 colab to predict the structure of dimeric MCAK, however we were not successful due to the large low complexity regions (Mirdita et al, 2021). Similarly, much of Kif18b, apart from the catalytic domain and the dimerization domain are made of low complexity regions (Fig S1c). These unstructured regions make it difficult to identify regions of interactions between MCAK and Kif18b.

To probe for the interaction between MCAK and Kif18b we incubated Kif18b-His bound to Nickel^2+^-agarose beads with purified SNAP-MCAK_1-177_ or SNAP-MCAK_584-725_. MCAK did not bind to Kif18b in solution (Fig S1d, e). Thus we hypothesized Kif18b may interact dynamically with MCAK only in the context of the microtubule, facilitated by EB proteins that bind both Kif18b and MCAK (Honnappa et al., 2009; Montenegro Gouveia et al., 2010; Stout et al., 2011). We first tested whether Kif18b interacts with EB proteins using GMPCPP-stabilized microtubules. EB3 alone does not strongly bind to GMPCPP microtubules (Maurer et al., 2011; Roth et al., 2018). Using *in vitro* reconstitution and total internal reflection fluorescence (TIRF) microscopy imaging, we imaged microtubules (rhodamine, Hilyte 647), Kif18b (GFP, mRuby3), EB3 (SNAP_647_-EB3) and MCAK (GFP) in the presence of 2 mM ATP. The polarity of the microtubules could be inferred because Kif18b is a microtubule plus end directed motor that accumulates at microtubule plus ends (McHugh et al., 2018). The amount of Kif18b accumulation at microtubule plus ends was length-dependent (Fig S2a). Recombinant SNAP_647_-EB3 alone uniformly and weakly decorated the GMPCPP-stabilized microtubule lattice (Fig 1c, d), and soluble SNAP_647_-EB3 provided some background fluorescence. Upon addition of 12.5 nM Kif18b, we observed that SNAP_647_-EB3 strongly co-localized with Kif18b at the plus end of microtubules (Fig 1c, d, S2b). Thus, on GMPCPP-stabilized microtubules Kif18b binds EB3 and enhances the localization of EB3 to the plus end.

Based on the Kif18b-dependent localization of MCAK to microtubule plus ends in HeLa cells (Fig 1a, b), we tested whether Kif18b influences the localization of MCAK on microtubules *in vitro*. Purified 12.5 nM MCAK-GFP bound diffusely along the length of double-stabilized (taxol and GMPCPP) microtubules (Fig 1e, f). With the addition of 12.5 nM Kif18b-mRuby3, the localization of MCAK shifted towards the plus end of the microtubule, although MCAK still decorated the lattice (Fig 1e, f). The localization of Kif18b-mRuby3 to the microtubule plus end was slightly reduced by the addition of MCAK and EB3 (Fig S2c). This could be due to the Kif18b C terminus engaging with EB3 and MCAK rather than the microtubule lattice. Indeed we previously showed the last 90 amino acids of Kif18b containing 2 SXIP motifs increase the residency time of Kif18b at microtubule plus ends threefold (McHugh et al., 2018). Thus, in our assay, Kif18b promotes the accumulation of both MCAK and EB3 proteins individually to the plus ends of microtubules. In the presence of both 50 nM SNAP_647_-EB3 and 12.5 nM Kif18b-GFP, MCAK plus end accumulation was further enhanced (Fig 1e, f), indicating while MCAK interacts weakly with EB proteins (Montenegro Gouveia et al., 2010) and Kif18b (Fig 1e, f), multivalent weak interactions of MCAK with both EB3 and Kif18b increase its accumulation to microtubule ends. The distribution of MCAK at the plus ends of microtubules varied upon addition of Kif18b or Kif18b + EB3, possibly reflecting different degrees of taper at the plus end of the taxol and GMPCCP-stabilized microtubule induced by depolymerases (MCAK/Kif18b motor domain) and microtubule stabilizing factors (Kif18b C terminus/EB3) (McHugh et al., 2018; Montenegro Gouveia et al., 2010).

To test if the Kif18b-dependent localization of MCAK was specific, we mixed the microtubule crosslinker PRC1-GFP (Subramanian et al., 2010) and Kif18b and incubated them with stabilized microtubules pre-immobilized on coverslips (Fig S2d, e). No accumulation of PRC1 at the ends of single microtubules was observed, confirming Kif18b-dependent delivery of MCAK and EB3 was specific. The presence of Kif18b at the ends of microtubules resulted in a small decrease in PRC1 intensity (Fig S2d), perhaps due to competition for space on the lattice in this region. Our data indicate that Kif18b promotes EB3 and MCAK plus end targeting and that Kif18b and EB3 proteins function cooperatively to facilitate MCAK microtubule plus end accumulation.

### Kif18b targets the N terminus of MCAK to microtubule plus ends

**Figure 2:**
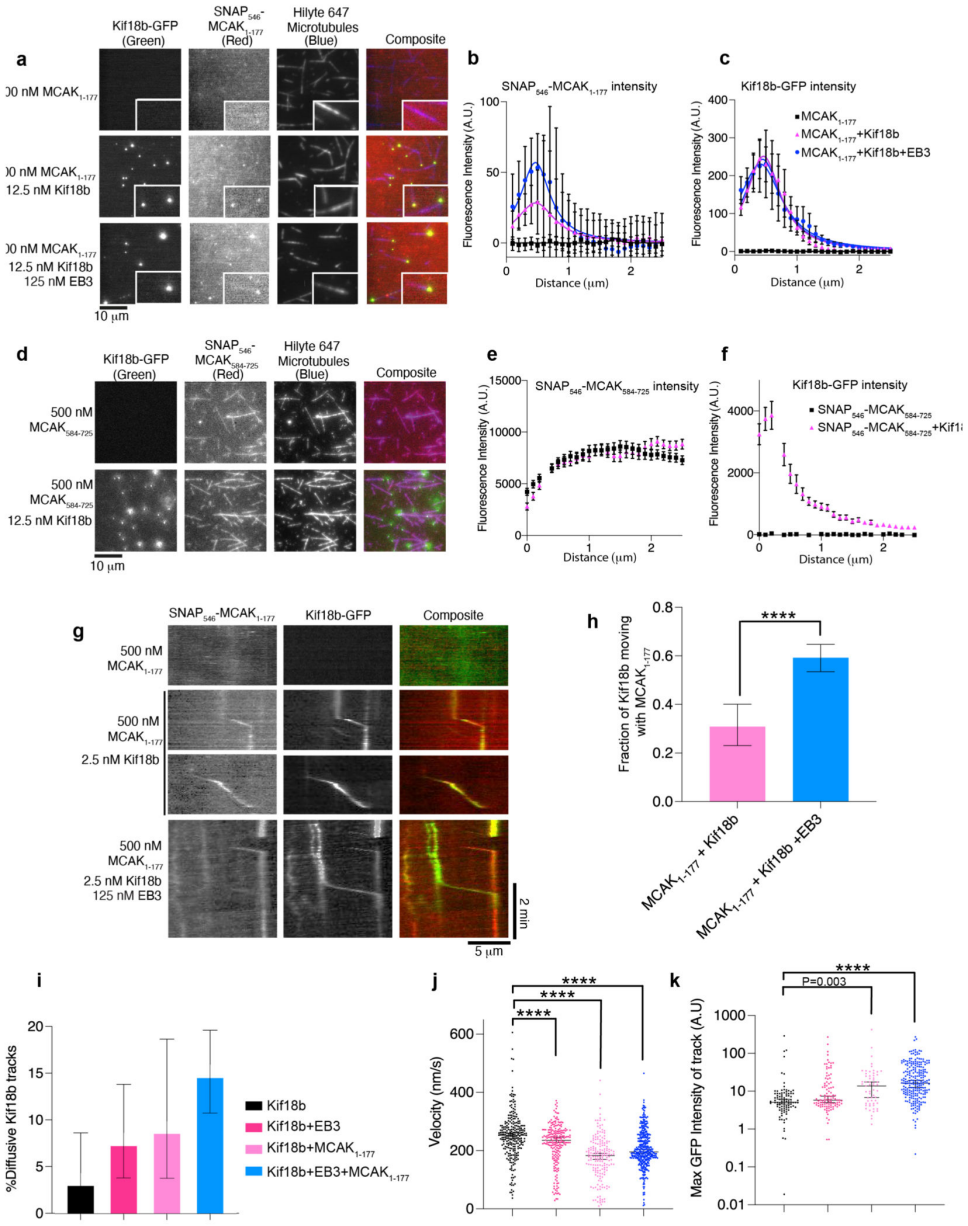
Single MCAK motors show increased lattice residency and directional movement in the presence of Kif18b. a) Representative images of Kif18b-GFP, SNAP_546_-MCAK_1-177_, and unlabeled EB3 on GMPCPP-stabilized HiLyte_647_-labeled microtubules. Scale Bar, 10 μm. b) Quantification of SNAP_546_-MCAK_1-177_ fluorescence intensity at the microtubule plus end (alone, black n=36), in the presence of 12.5 nM Kif18b-GFP (pink, n=43) and 125 nM EB3 (blue, n=28) (mean and S.E). Kruskal-Wallis test at 0.5 μm, MCAK versus Kif18b + MCAK, *** P=0.0001, MCAK versus Kif18b + MCAK + EB3, **** P<0.0001. c) Quantification of GFP fluorescence intensity at the microtubule plus end when MCAK alone is present (black, n=36), in the presence of 25 nM Kif18b-GFP (pink, n=43), and 125 nM EB3 + 12.5 nM Kif18b-GFP (blue, n=28) (mean and S.E). d) Representative images of Kif18b-GFP and SNAP_546_-MCAK_584-725_ on GMPCPP-stabilized HiLyte_647_-labeled microtubules. Scale Bar, 10 μm. e) Quantification of SNAP_546_-MCAK_584-725_ fluorescence intensity at the microtubule plus end (alone, black n=57) and in the presence of 12.5 nM Kif18b-GFP (pink, n=54, mean and S.E). f) Quantification of GFP fluorescence intensity at the microtubule plus end when MCAK alone is present (black, n=57), or in the presence of 12.5 nM Kif18b-GFP (pink, n=54, mean and S.E). g) Kymographs of 500 nM SNAP_546_-MCAK_1-177_ (red) alone, and with 2.5 nM Kif18b-GFP (green) in the presence or absence of 125 nM EB3 on GMPCPP-stabilized microtubules. Scale Bar 5 μm (horizontal) and 2 min (vertical). h) Fraction of moving particles in the Kif18b-GFP channel which co-localize with particles moving in the SNAP_546_-MCAK_1-177_ channel with 500 nM MCAK_1-177_ and 2.5 nM Kif18b-GFP and with (n=110) and without (n=287) 250 nM EB3 (error bars represent the Wilson/Brown 95% C.I.). Asterisks indicate Fishers exact test significance value. ****P<0.0001. i) Bar graph showing fraction of Kif18b tracks displaying diffusive behavior when Kif18b is alone (black, n=98), in the presence of EB3 (magenta, n=109), MCAK_1-177_ (pink, n=58) and EB3/MCAK_1-177_ (blue, n=246). Error bars represent the Wilson/Brown 95% confidence interval. j) The average velocity (mean and S.E.M.) of Kif18b-GFP motors for Kif18b-GFP alone (n=252), Kif18b-GFP+EB3 (n=216), Kif18b-GFP and SNAP_546_-MCAK_1-177_ with and without 125 nM EB3 (n=372 and n=178). Asterisks indicate ordinary One-way Anova test significance value. ****P<0.0001. k) Scatter dot plot showing the distribution of GFP fluorescence intensity of Kif18b binding events for Kif18b alone (n=98), Kif18b + EB3 (n=107), Kif18b + MCAK_1-177_ (n=59) and Kif18b + EB3 + MCAK_1-177_ (n=247). Bars represent the median and 95% confidence interval. Asterisks indicate KS test significance value. *** P=0.0003, ****P<0.0001

Previous work showed that in cells, Kif18b does not interact with a phosphomimetic mutant of MCAK, when sites phosphorylated by Aurora kinases in the N terminus of MCAK were mutated to glutamates (Tanenbaum et al., 2011). Thus, we hypothesized the interaction with Kif18b was through the N terminus of MCAK. To test this, we expressed the N terminus of MCAK_1-177_ and C terminus of MCAK_584-725_ fused to SNAP labelled with a 546 fluorophore. Alone SNAP_546_-MCAK_1-177_ did not bind to the microtubules nor did it bind Kif18b in solution (Fig 2a-c, S1e). In the presence of 12.5 nM Kif18b we observed an enrichment of SNAP_546_-MCAK_1-177_ at the plus ends of GMPCPP-stabilized microtubules, where Kif18b accumulates (Fig 2a-c). This was further enhanced by the addition of EB3. In contrast, the C terminus of MCAK_584-725_ uniformly decorated the microtubule lattice both in the presence and absence of Kif18b (Fig 2d-f). These results indicate microtubule-bound Kif18b interacts specifically with the N terminus of MCAK.

### Kif18b increases directional MCAK movement along the lattice

We next tested whether Kif18b can transport MCAK along microtubules using SNAP_546_-MCAK_1-177_. We observed SNAP_546_-MCAK_1-177_ moving with Kif18b-GFP along GMPCPP-stabilized microtubules in the absence and presence of unlabeled EB3 by two-colour fluorescence imaging (Fig 2g). A fraction of Kif18b motors displayed co-localization with the N termini molecules of MCAK. This was increased in the presence of EB3, which interacts both with MCAK and Kif18b. In the presence of EB3, most Kif18b motors co-localized with MCAK N termini molecules (Fig 2h). Kif18b displayed a modest increase in diffusive behavior in the presence of EB3 and/or MCAK N terminus, indicating that binding of cargos impacts the behavior of Kif18b on the lattice (Fig 2i). Additionally, the velocity of Kif18b decreased when bound to the N terminus of MCAK and/or EB3 (Fig 2j). Kif18b-GFP remains dimeric on microtubules when alone or in the presence of EB3 (Fig 2k, Fig S3a, b). However, there is an increase in fluorescence intensity of Kif18b-GFP moving on microtubules in the presence of the MCAK N terminus, indicating potential oligomerization (Fig 2k). Together, our work indicates that Kif18b interacts with MCAK through its N terminus and this interaction is further stabilized by EB3, which binds both Kif18b and MCAK. Kif18b transports the N terminus of MCAK towards the plus end of the microtubule lattice, while MCAK also influences the motile properties of Kif18b.

To test whether Kif18b can promote transport of full-length MCAK, we then imaged low concentrations of labeled MCAK-GFP on stable microtubules to record single molecule events using fast imaging (10 frames per second) (Fig S3c). MCAK alone diffused on microtubules, we measured a diffusion coefficient of 5510±734 nm^2^/s (Fig S3d), and remained bound to the lattice for 1.3±0.5 seconds on average (Fig S3e). In the presence of 10 nM Kif18b, MCAK lattice residency increased to 2.4±0.2 seconds and MCAK was more diffusive with a 4-fold increase in diffusion coefficient to 24082±1478 nm^2^/s (Fig S3c-e). At 10 nM Kif18b and subnanomolar concentrations of MCAK-GFP, required for single molecule imaging, we saw MCAK accumulating at the microtubule ends. While we cannot distinguish the polarity of the stable microtubule, we can infer that MCAK is accumulating at microtubule plus ends, where the Kif18b concentration will be at its highest, given the plus end accumulation of Kif18b (Fig S3c) (McHugh et al., 2018). We also observed events of directional movement of MCAK, often at rates of around 300 nm/s corresponding to those seen for single Kif18b motors (McHugh et al., 2018) (Fig S3c). This when combined with our data for SNAP_546_-MCAK_1-177_ (Fig 2g) indicates that the accumulation of MCAK at the plus ends of microtubules in the presence of Kif18b is due, at least in part, to direct plus end directed transport of MCAK as a cargo of Kif18b. Multivalent interactions between Kif18b, EB3 and MCAK proteins in the context of the microtubule create an Kif18b-EB3-MCAK plus end-localized network.

### The C terminus of Kif18b stabilizes microtubules

**Figure 3:**
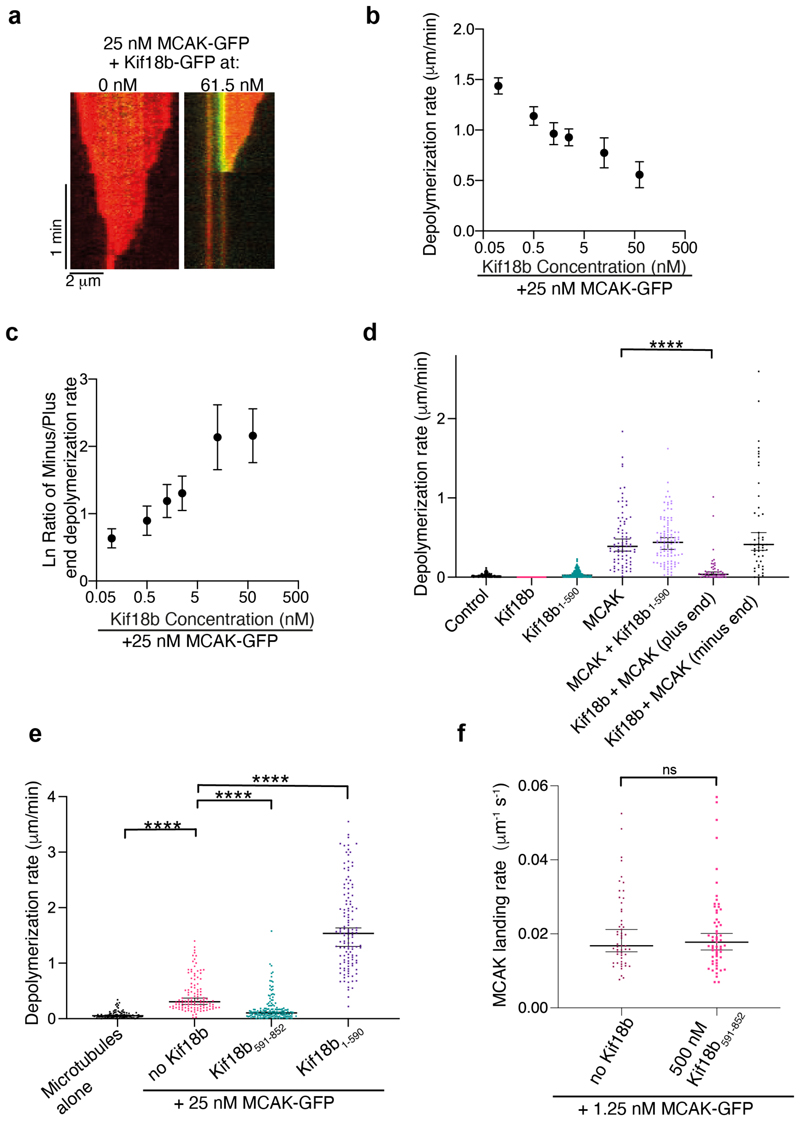
The C-terminal tail of Kif18b opposes MCAK mediated microtubule depolymerization. a) Example kymographs showing the depolymerization of GMPCPP microtubules by 25 nM MCAK-GFP in the presence (right) and absence (left) of 61.5 nM Kif18b-GFP. Scale bars 1 min (vertical) and 2 μm (horizontal) b) Increasing Kif18b-GFP concentrations lead to lower microtubule depolymerization rates mediated 25 nM MCAK-GFP. Mean and S.E., n=270. Spearman correlation of -1 with a P-value of 0.004 (***), showing a monotonic relationship between microtubule depolymerization rate and Kif18b concentration. c) Ratio of minus/plus end depolymerization rate with increasing Kif18b concentration in the presence of 25 nM MCAK-GFP. Mean and S.E., n = 270. Spearman correlation of +1 with a P-value of 0.004 (***), showing a monotonic relationship between ratio of minus/plus end depolymerization rate and Kif18b concentration. d) Depolymerization rates for GMPCPP stable microtubules alone (n=110), with 25 nM Kif18b-GFP (n=74), 25 nM Kif18b_1-590_-GFP (n=272), 25 nM MCAK-GFP (n=82), 25 nM Kif18b_1-590_-GFP + 25 nM MCAK-GFP (n=104) and 25 nM Kif18b-GFP + 25 nM MCAK-GFP (plus and minus end rates) (n=49). Bars represent the median and 95% confidence intervals. Asterisks indicate an ordinary Kruskal-Wallis test significance value when comparing depolymerization rates in the presence of MCAK with MCAK+Kif18b constructs. ****P<0.0001. (Also see Table S1). e) Depolymerization rates for GMPCPP stable microtubules alone (n=94) with 25 nM MCAK (n=113), 25 nM MCAK + 250 nM Kif18b_1-590_-GFP (absolute concentration of 500 nM) and 25 nM MCAK + 500nM Kif18b_591-852_-GFP (n=165 and 127) Asterisks indicate an ordinary Kruskal-Wallis test significance values ****P<0.0001. f) MCAK landing rate on microtubules with 1.25 nM MCAK-GFP in the presence or absence of 500nM Kif18b_591-852_ (n=53 and 59 respectively) (Table S1). Bars represent the median and 95% confidence intervals. Kolmogorov-Smirnov test, P=0.9754.

We previously showed that the C terminus of Kif18b binds to microtubules and enables Kif18b to remain bound to the plus ends of both stable and dynamic microtubules (McHugh et al., 2018). It also opposes the weak depolymerase activity of Kif18b similarly to budding yeast Kinesin-8 Kip3 (Su et al., 2011). We therefore tested whether Kif18b interfered with MCAK-induced depolymerization of stable microtubules through its microtubule-binding C terminus. It is important to note that full-length Kif18b-GFP accumulates at microtubule plus ends but does not depolymerize GMPCPP-stabilized microtubules and that MCAK causes slow microtubule depolymerization of GMPCPP-stabilized microtubules from both ends rather than inducing catastrophe (Fig 3a). This slow depolymerization of microtubules allowed us to probe how MCAK and Kif18b can work together at microtubule ends (Fig 3a-e) and image single molecules of MCAK on microtubules (Fig 3f). In the presence of 25 nM MCAK-GFP, addition of Kif18b caused a decrease in microtubule end depolymerization dependent on Kif18b concentration and specific to the plus end where Kif18b-GFP accumulated (Fig 3a, b). This led to an asymmetry in plus and minus end depolymerization rates, proportional to the concentration of Kif18b (Fig 3c). In the presence of 25 nM MCAK and 25 nM full-length Kif18b, the rate of the microtubule plus end depolymerization was severely reduced to 0.036 μm/min while the free minus end depolymerization rate was similar to that with MCAK alone (0.390 μm/min) (Fig 3d, Table S1). We found that in the presence of MCAK and a C-terminally truncated Kif18b_1−590_-GFP, the microtubule depolymerization rate was similar to the rate in the presence of MCAK alone, indicating the plus end localization of the C terminus of Kif18b interfered with MCAK-mediated depolymerization of stable microtubules. We then used a high concentration of Kif18b truncation mutants to compensate for their inability to accumulate at microtubule plus ends (Fig 3e). 250 nM Kif18b_1-590_ displayed strong depolymerase activity, as previously shown (McHugh et al., 2018). 500 nM Kif18b_591-852_ (which is monomeric and corresponds to the concentration of molecules for 250 nM dimeric Kif18b) significantly reduced the rate of MCAK-induced depolymerization, without affecting the landing rate of MCAK (Fig 3f). This indicates the C terminus of Kif18b further stabilizes the GMPCCP-stabilized microtubule lattice against MCAK depolymerase activity rather than preventing MCAK association with the microtubule.

On dynamic microtubules, 500 nM of the C terminus of Kif18b, increased the growth rate of microtubules (Fig S4a), consistent with a role in microtubule lattice stabilization. However, it did not impact the catastrophe frequency or overall microtubule length (Fig S4b, c). In the presence of 12.5 nM MCAK and 500 nM Kif18b_591-852_ the growth rate of microtubules remains elevated (Fig S4d) and the catastrophe frequency and overall microtubule length were largely unchanged (Fig S4e, f). Thus, in the context of dynamic microtubules the C terminus of Kif18b alone does not stabilize microtubules against MCAK activity. Overall, stabilized microtubules allow the accumulation of Kif18b and MCAK at microtubule plus ends where Kif18b mediates a stabilizing effect. In contrast on dynamic microtubules, catastrophe led to loss of motors associated with the depolymerizing end, lessening any stabilizing role the C terminus of Kif18b may have. Most kinesin-8 appear to use their C terminal domain to fine tune their activities at the expense of their microtubule depolymerizing motor domain, but most importantly facilitate their plus end targeting (Stumpff et al., 2011; Su et al., 2013; Su et al., 2011).

### Effect of Kif18b, EB3 and MCAK on microtubule dynamics

**Figure 4:**
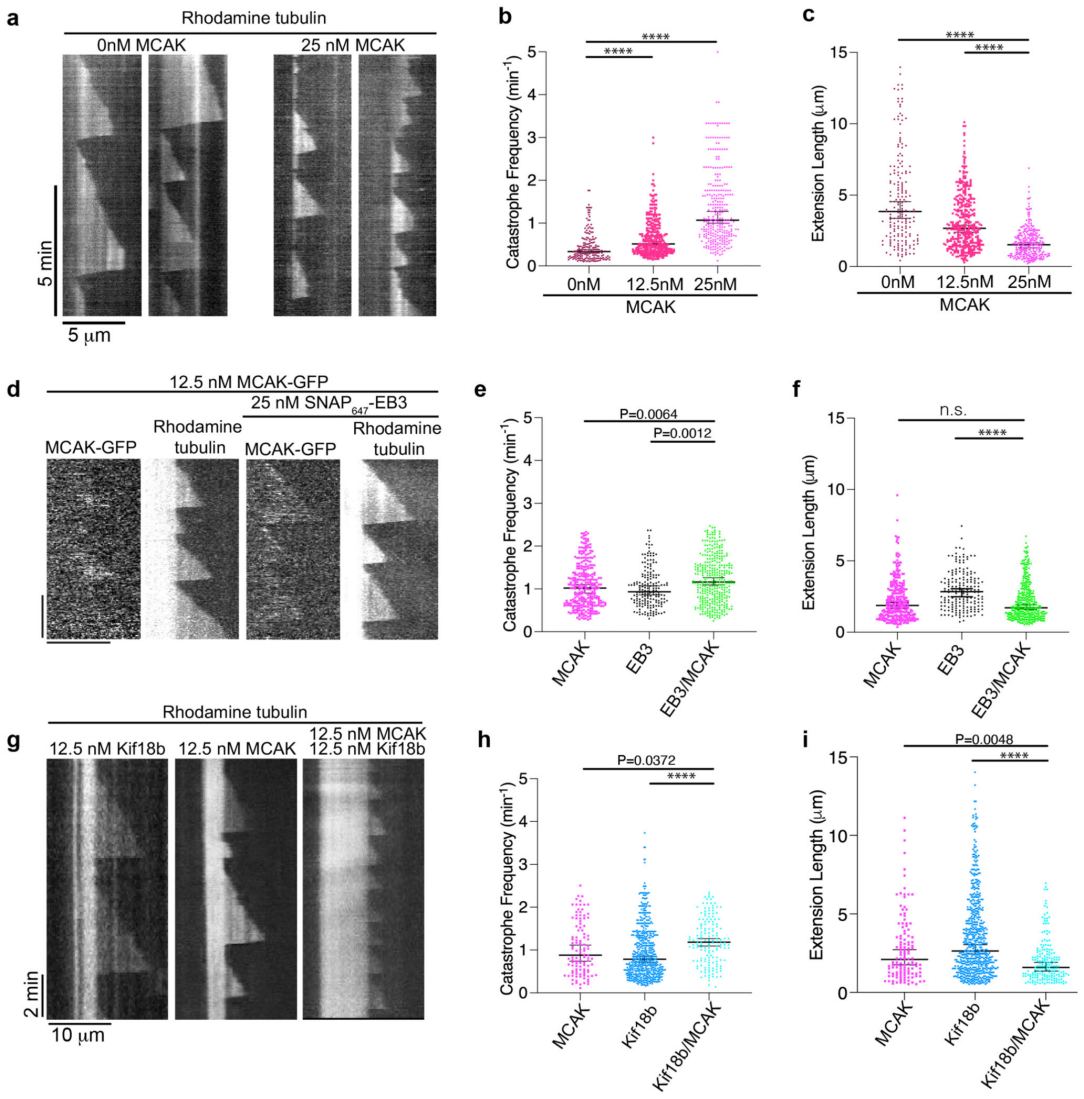
MCAK and Kif18b increase microtubule catastrophe frequency and decrease microtubule length. a) Representative kymographs of dynamic microtubules in the presence of 0 nM and 25 nM MCAK-GFP with 12 μM tubulin. Scale 2 mins (vertical) and 10 μm (horizontal) b) Microtubule catastrophe frequency and c) length of dynamic microtubule extensions in the presence of increasing MCAK concentration. n > 184 for each condition, values (Median and 95% C.I.) are given in Table S1. We were unable to consistently detect microtubule growth events that occurred for a less than 25 seconds, so these data were removed from our analysis. Asterisks indicate Kruskal-Wallis test significance values, **** P<0.0001. d) Representative kymographs of dynamic microtubules in the presence of 12.5 nM MCAK-GFP and 25 nM SNAP_647_-EB3 with 7 μM tubulin. Scale 10 μm (horizontal) and 2 mins (vertical) e) Microtubule catastrophe frequency and f) length of dynamic microtubule extensions in the presence of 12.5 nM MCAK and 25 nM SNAP_647_-EB3. n > 175 for all conditions, values (Median and 95% C.I.) are given in Table S1. Asterisks indicate Kruskal-Wallis test significance values, **** P<0.0001. g) Representative kymographs of dynamic microtubules in the presence of 12.5 nM MCAK-GFP or/and 12.5 nM Kif18b-mRuby3 with 7 μM tubulin. Scale, 2 mins (vertical) and 10 μm (horizontal) h) Microtubule catastrophe frequency and i) length of dynamic microtubule extensions in the presence of 12.5 nM MCAK-GFP or/and 12.5 nM Kif18b-mRuby3. n > 110 for all conditions, values (Median and 95% C.I.) are given in Table S1. Asterisks indicate Kruskal-Wallis test significance values, **** P<0.0001.

We therefore sought to define the properties of the Kif18b-MCAK-EB3 network on dynamic microtubules. We first analyzed the effects of MCAK alone on dynamic microtubules using GMPCPP-stabilized seeds with microtubules growing off them. MCAK acts as a strong catastrophe factor (Fig 4a-c, Table S1). Increasing MCAK concentration led to high catastrophe frequencies, ranging from 0.34 min^-1^ to 1.1 min^-1^ for 0 to 25 nM (Fig 4a, b, Table S1) as previously reported by (Gardner et. al. 2011). The length of microtubules extensions was significantly shorter with increasing concentrations of MCAK with 2.68±0.21 and 1.51±0.14 μm in the presence of 12.5 and 25 nM MCAK respectively, while microtubules extensions alone were 3.85±0.69 μm long (median±95% C.I.) (Figure 4c, Table S1).

We next analyzed whether microtubule dynamics in the presence of MCAK were altered by the addition of EB3 (Fig 4d-f). When using 12.5 nM MCAK, catastrophe frequency increased significantly from 1.02 to 1.16 min^-1^ upon addition of 25 nM EB3 (Fig 4e). However, there were no major changes in microtubule length between conditions where MCAK or MCAK and EB3 were used (Fig 4f, Table S1). In addition, we could see little enrichment of MCAK at microtubule plus ends under our conditions (Fig 4d). This is explained by the dissociation constant in the micromolar range of EB proteins with MCAK of 10 μM (Buey et al., 2012). It was however possible to see robust EB-dependent MCAK-tracking the growing ends of microtubules when higher concentrations of EB3 and MCAK were used. However, the microtubule seeds had to be additionally stabilized with taxol to counteract MCAK depolymerase activity as in (Montenegro Gouveia et al., 2010) and (Fig S5a). Thus, we did not quantify microtubule dynamics under these conditions. In total these data indicate at low nanomolar concentration, MCAK depolymerizes microtubules independently of EB3.

We next tested how Kif18b and MCAK influence microtubule dynamics when combined. We have previously shown that Kif18b has only a modest effect on microtubule dynamics, increasing catastrophe frequency and growth rate, leading to a slightly reduced microtubule extension length (McHugh et al., 2018). Using 12.5 nM of each dimeric motor, we compared the effect of the motors alone and in combination (Fig 4g). 12.5 nM MCAK increased catastrophe frequency (Fig 4h, Table S1) and this occurred to a greater extent than for Kif18b (Fig 4g, i,). In the presence of both MCAK and Kif18b, catastrophe frequency further increased (Fig 4i). While microtubule length was reduced when both Kif18b and MCAK were present (Figure 4g, i).

### Kif18b, EB3 and MCAK track the growing ends microtubules together to increase catastrophe frequency and decrease microtubule length

**Figure 5:**
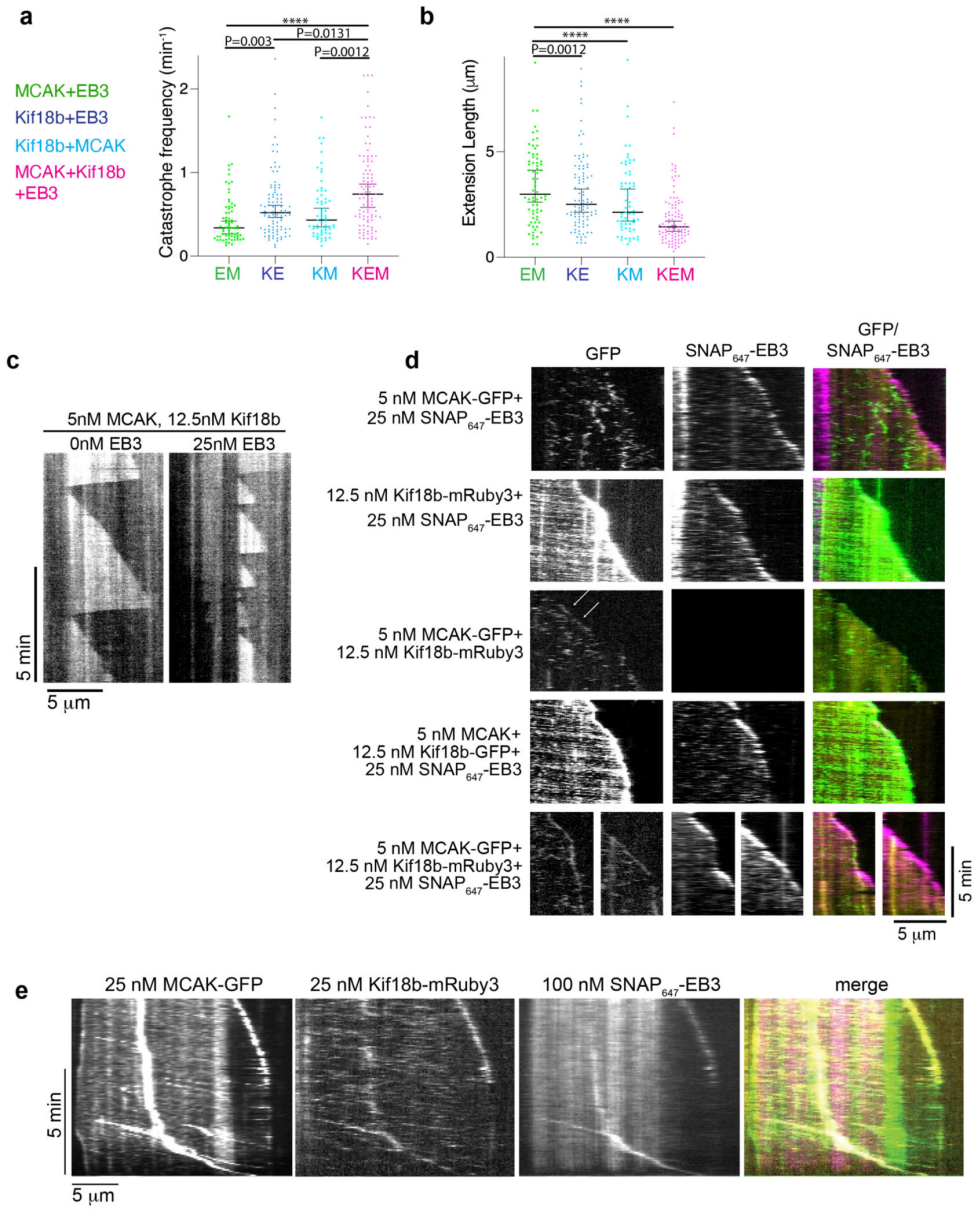
Kif18b-depedent delivery of MCAK and EB3 to microtubule plus ends leads to high catastrophe frequencies and shortening of microtubule length. a) Microtubule catastrophe frequency and b) length of dynamic microtubule extensions in the presence of 5 nM MCAK-GFP and 12.5 nM Kif18b-mRuby3 and 25 nM SNAP_647_-EB3. n values, median and 95% C.I. are given in Table S1. Asterisks indicate Kruskal-Wallis test significance values, **** P<0.0001 c) Representative kymographs of dynamic microtubules, in the presence of 5 nM MCAK-GFP and 12.5 nM Kif18b-mRuby3 with 0 nM or 25 nM SNAP_647_-EB3 with 12 μM tubulin. Scale, 5 mins (vertical) and 5 μm (horizontal). d) Representative kymographs of dynamic microtubules in the presence of 5 nM MCAK-GFP or His-MCAK, 12.5 nM Kif18b-mRuby3 or -GFP and 25 nM SNAP_647_-EB3 with 12 μM tubulin. Scale, 5 mins (vertical) and 5 μm (horizontal). Seeds were stabilized with GMPCPP and taxol, the seeds were washed with 10 volumes of BRB80 before 12 μM free tubulin and protein mix was added, note residual taxol stabilizes the lattice. e) Representative kymographs of dynamic microtubules in the presence of 25 nM MCAK-GFP, 25 nM Kif18b-mRuby3 and 100 nM SNAP_647_-EB3 with 12 μM tubulin. Scale, 5 mins (vertical) and 5 μm (horizontal). Seeds were stabilized with GMPCPP and taxol, the seeds were washed with 5 volumes of BRB80 before 12 μM free tubulin and protein mix was added, note residual taxol stabilizes the lattice.

We next defined the properties of the Kif18b-EB3-MCAK network on dynamic microtubules. When using 12.5 nM MCAK, 12.5 nM Kif18b and 25 nM EB3, no dynamic microtubule extensions were seen (Figure S5b), indicating the combination of Kif18b, EB3 and MCAK had a dramatic effect on microtubules, which could not equate to the sum of their individual activities. To enable quantification of this effect, we decreased the amount of MCAK in our assays to 5 nM, while keeping 12.5 nM Kif18b and 25 nM EB3 and imaged the microtubule extensions grown from GMPCPP stabilized seeds in the presence 12 μM tubulin. A significant effect was seen in the presence of all three proteins on both catastrophe frequency and microtubule extension length. Catastrophe frequency was the highest and conversely the dynamic microtubule extensions were the shortest under these conditions (Figure 5a, b). There was a 2-fold increase in catastrophe frequency from 0.34 min^-1^ for MCAK+EB3 to 0.74 min^-1^ for MCAK+EB3+Kif18b (Fig 5a). Overall, this resulted in a 1.8-fold reduction in the length of dynamic extensions when Kif18b was added to EB3+MCAK with microtubules remaining dynamic and short (Fig 5b, c). We then imaged the three proteins simultaneously using taxol and GMPCPP double-stabilized microtubule seeds prepared as in (Montenegro Gouveia et al., 2010) (Figure 5d). As expected, MCAK mostly diffused on the lattice while EB3 tracked the growing ends of microtubules and Kif18b exhibited processive behavior walking towards microtubule plus ends. In the presence of Kif18b, there was a slight enrichment of MCAK towards the growing plus end of microtubules (White arrows) and plus end directed processive events could be seen with MCAK-GFP moving at 296 nm/s similar to that we previously reported for Kif18b alone (Fig S5c-d) (McHugh et al., 2018). When all three proteins, Kif18b, MCAK and EB3 were present, MCAK tracked the plus ends of growing microtubules along with Kif18b and EB3 (Fig 5d). Addition of EB3 increased the proportion of processive plus-end directed MCAK-GFP motors, although the majority, >90%, of MCAK-microtubule binding events were diffusive (Fig S5e). When the protein concentration was slightly higher, Kif18b-mediated transport events of EB3 and MCAK towards the microtubule end were more easily apparent (Figure 5e, movie 1). These complexes formed bright clusters of multiple proteins indicating assembly of the Kif18b-EB3-MCAK network and were transported to the plus ends by Kif18b. Together, the Kif18b-EB3-MCAK network cooperates in an integrated manner to dramatically increase microtubule catastrophe in mitosis.

## Discussion

Microtubule dynamics dramatically increase at mitotic onset when microtubules shorten to remodel the microtubule cytoskeleton and assemble a bipolar spindle (Belmont et al., 1990; Piehl and Cassimeris, 2003). This change in microtubule length is attributed mainly to an increase in the catastrophe frequency (Belmont et al., 1990). While Kif18b alone is a weak microtubule depolymerase *in vitro*, it is a major microtubule depolymerization factor in mitosis once released from the nucleus (Lee et al., 2010). In the absence of Kif18b or MCAK, mitotic microtubules remain long, leading to spindle assembly and positioning defects in humans (McHugh et al., 2018; Rankin and Wordeman, 2010; Stout et al., 2011; Tanenbaum et al., 2011; van Heesbeen et al., 2016). Here we show mechanistically how MCAK, Kif18b and EB3 cooperate to increase the catastrophe frequency of microtubules. Using single molecule imaging and *in vitro* reconstitution we find that Kif18b can transport MCAK via interactions with both the MCAK N terminus and EB3 to microtubule plus ends where they promote microtubule catastrophe. It is clear from our data that missing any one component of the MCAK-EB3-Kif18b network results in reduced capability for limiting microtubule length.

Our data challenge previous models that postulate that MCAK uses diffusion alone to reach microtubule ends (Cooper et al., 2010; Helenius et al., 2006). While 1-dimensional diffusion on the microtubule lattice is an efficient way to reach a microtubule end over short distances, diffusion is not necessarily an efficient way to reach specifically the plus end of a microtubule in a cell. The diffusion rate of MCAK was calculated as 5510 nm^2^/s with a residency time of 1.3 seconds. This would mean that a single motor on average explores just 84 nm of the microtubule lattice with each encounter. In the presence of Kif18b, both MCAK diffusion and residency time increase: in the presence of Kif18b, MCAK would diffuse an average of 240 nm, thereby increasing the probability MCAK would reach or get close to a microtubule plus end. However, diffusion alone does not explain how MCAK localizes primarily to microtubule plus ends on crowded and long microtubules in cells.

The molecular structure of the MCAK-Kif18b-EB network remains poorly understood. MCAK binding to both Kif18b and EB proteins is weak (Fig S1e)(Buey et al., 2012). Given Kif18b and MCAK have large regions of low complexity (Fig S1b, c) it is likely they assemble as higher oligomers through weak multivalent interactions within their unstructured domains on the microtubule (Fig 5e), offering many binding sites to EB3.

**Figure 6:**
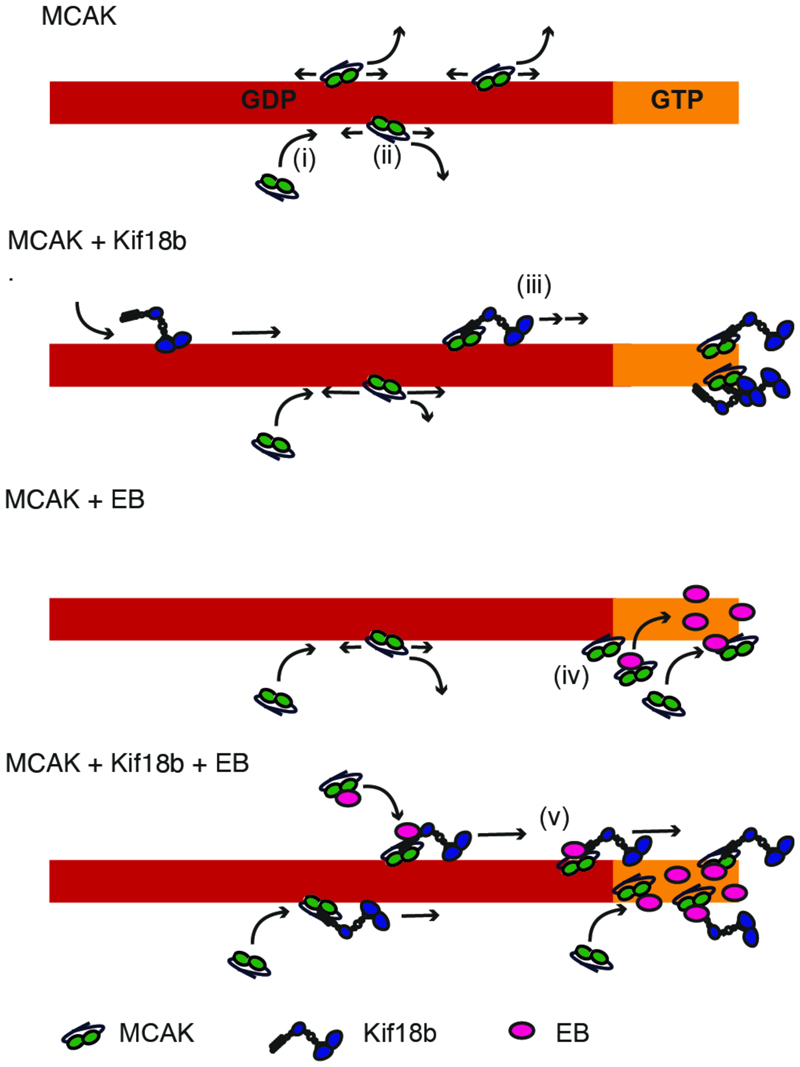
Model of the Kif18b-EB-MCAK network at dynamic microtubule ends. MCAK alone binds to the microtubule lattice (i) and diffuses for short distances before detaching (ii). Few MCAK motors reach the microtubule end. In the presence of Kif18b, MCAK transport occurs (iii). This leads to more MCAK arriving at the microtubule ends. (iv) MCAK also binds to EB proteins, that are binding and exchanging at the GTP cap of microtubules. This enables the accumulation of MCAK to microtubule plus ends (iv). When both EB and Kif18b proteins are present, MCAK can bind EB and Kif18b at the microtubule plus ends or form a tripartite complex that can be transported to microtubule ends by Kif18b. As all proteins are dimeric, they can form a network of interactions at microtubule plus ends where they accumulate and promote microtubule depolymerization (v).

The localization of endogenous MCAK at microtubule plus ends in interphase cells is barely detectable using immunofluorescence, prompting most to use fluorescently labelled MCAK (Moore et al., 2005). Endogenous MCAK has only been observed in mitosis and is dependent on Kif18b and EB proteins (Figure 1) (Tanenbaum et al., 2011), suggesting less MCAK is present at the ends of interphase microtubules. Indeed, similarly to Kif18b, the most striking effects of Kinesin-13 are on mitotic microtubules. Our work demonstrates that Kif18b, EB3 and MCAK collectively form a protein network at dynamic microtubule plus ends at very low concentration of each component. Here Kif18b plays a critical role as an integration platform to modulate the depolymerase activity of the network. Kif18b enhances the accumulation of MCAK on the lattice and at microtubule ends and delivers MCAK and EB3 proteins to microtubule ends (Figure 6), where they work together in an integrated manner to increase microtubule catastrophe. The unique and potent properties of the network cannot be imparted to the sum of the individual components (Fig 5).

Two distinct populations of Kif18b decorating astral microtubule plus ends have been reported; one population of Kif18b colocalizes with EB1 while the other precedes EB1 at the plus end of the microtubule (Shin et al., 2015). Kif18b is a fast motor, with a speed of 350 nm/s *in vitro* and up to 700 nm/s *in vivo* (McHugh et al., 2018; Tanenbaum et al., 2014), faster than microtubule growth speed around 200-250 nm/s (Sironi et al., 2011). Thus Kif18b can reach the growing end of a microtubule and accumulate there using its C-terminal microtubule-binding tail independently of EB proteins (McHugh et al., 2018). Indeed a small region at the growing end of microtubule is EB-free (Maurer et al., 2014) and lacks lateral stabilization of protofilaments (McIntosh et al., 2018). It is possible that Kif18b brings MCAK and EB3 to the terminal tubulin dimers of microtubules where tubulin is assembled into single protofilaments but not yet as a closed microtubule, to promote depolymerization. There, the Kif18b-MCAK-EB3 complex could efficiently depolymerize microtubule growing ends before they have been stabilized through lateral contacts of protofilaments, to avoid an additional energy barrier to depolymerization.

Importantly, each interaction within this Kif18b-MCAK-EB3 network is individually regulated by mitotic phosphorylation, a rapid and reversible post-translational modification, while Kif18b is confined to the nucleus before mitosis and irreversibly degraded at the end of mitosis. Thus, the integrity and activity of this depolymerizing network is tightly regulated to allow for spatial, local and temporal regulation of microtubule dynamics during mitosis. It would be interesting to define whether Kif18b also delivers other microtubule-associated proteins with low complexity domains to plus ends, through interactions with its unstructured C terminus. Finally, our work highlights the importance of studying motors not only in isolation but examining their collective behavior and the underlying emerging function. How cargos influence motor activity remains a poorly understood phenomenon. Rather than having a microtubule motor that provides transport to passive cargos, we reveal here how the three components of mitotic-specific Kif18b-EB-MCAK network, two of which are motors, act in an integrated manner to provide increased depolymerization of microtubule plus ends with temporal and spatial layers of control.

## Methods

### Cloning

The Sf9 insect cell expression constructs for producing full-length human Kif18b-GFP, Kif18b_1-590_-GFP and MCAK-GFP with a C-terminal 6xHis have been described elsewhere (McHugh et al., 2018; Talapatra et al., 2015). For Kif18b-mRuby3-His, Kif18b was cloned in to pFl-mRuby3-His using restriction enzymes. mRuby3 was synthesized after codon optimization for expression in insect cells. Kif18b_591-852_ was cloned in to pET3atr with an N-terminal 6xHis. MCAK_1-177_ and MCAK_584-725_ were cloned into vectors containing His-TEV-SNAP. EB3 was cloned into vectors containing a His, His-TEV-SNAP or His-GFP tag. The His-TEV-GFP-PRC1 plasmid was a gift from T. Kapoor and is described in (Subramanian et al., 2010).

### Cell culture

HeLa cells were used and maintained in DMEM (Lonza) supplemented at 37°C in a humidified atmosphere with 5% CO_2_. The generation of the Kif18b knockout cell line is described in (McHugh et al., 2018). Cells were purchased from ATCC and are monthly checked for mycoplasma contamination (MycoAlert detection kit, Lonza).

### Immunofluorescence and microscopy

Cells were washed in PBS and fixed in 3.8% formaldehyde in PHEM buffer (60 nM Pipes, 25 mM HEPES, 10 mM EGTA, 2 mM MgSO_4_, pH 7.0) for 10 minutes. Immunofluorescence in human cells was conducted using antibodies against mouse EB1 (BD transduction laboratories, 1:400) and a custom rabbit MCAK antibody (1:500-1000). The MCAK antibody, raised against GFP-MCAK, was generated by Eurogenetec using the Speedy program. Over time, the antibody became rapidly unstable and non-specific. Images were acquired using a DeltaVision core microscope (Applied Precision) with a 100x lens (NA: 1.4) equipped with a CoolSnap HQ2 CCD camera. 10-20 z-sections were acquired at 0.2-0.5 μm. Comet intensities were quantified as previously described (McHugh et al., 2018). Experiments were repeated three times independently.

### Protein expression and purification

Kif18b and MCAK proteins were expressed using a baculovirus expression system, Sf9 cells were infected with virus for each construct for 60-72 hours. His-Kif18b_591-852_, His-SNAP-MCAK_1-177_, His-SNAP-MCAK_584-725_, His-EB3 and His-GFP-PRC1 proteins with the his_6_ tag cleavable with the 3C protease were expressed in BL21 Codon+ *E. coli* cells overnight at 18°C. His-tagged Kif18b and MCAK proteins were purified as previously described (Talapatra et al., 2015). His-GFP-EB3, His-SNAP-EB3, and His-GFP-PRC1 proteins were purified using a HisTrap HP column and a gradient elution. His-SNAP-EB3, His-SNAP-MCAK_1-177_ and His-SNAP-MCAK_584-725_ were then coupled to SNAP-Cell 647-SiR dye or SNAP- Cell 546-SiR dye (NEB) by incubation at 37°C for 30 minutes or overnight at 4°C. Labelling of His-SNAP-EB3 was around 30%, while MCAK labelling with 80-90%. All proteins were further purified using gel filtration chromatography pre-equilibrated in gel filtration buffer (25 mM Pipes, pH 6.9, 150 mM NaCl, 300 mM KCl, 5 mM β-mercaptoethanol, 1 mM MgCl_2_, 1 mM Na-EGTA, and 1 mM ATP). Proteins were frozen in LN_2_ within 24 hours of cell lysis and stored for up to 3 months at -70°C (Kif18b, MCAK) or 6 months (EB3, PRC1). Protein concentration is reported for its oligomeric state in solution. Activity of frozen proteins was found to be consistent with results from freshly purified proteins within these timescales. Porcine brain tubulin was purified as described (Castoldi and Popov, 2003) and stored in liquid nitrogen long term.

### Sample preparation for TIRF microscopy

Samples were prepared as detailed in McHugh et al. 2018. An ATP concentration of 2 mM and a temperature of 30°C were used for all experiments. The stated concentrations of protein (or an equivalent volume of gel filtration buffer, up to a maximum of 3.5% total volume) were added to the final assay buffer (BRB80 with 2 mM ATP, 0.5 mg/ml casein, and an oxygen-scavenging system [0.2 mg/ml glucose oxidase, 0.035 mg/ml catalase, 4.5 mg/ml glucose, and 140 mM β-mercaptoethanol]), slides were sealed with Valap and imaged immediately. For dynamic microtubule experiments, the assay buffer also contained 7-12 μM tubulin (6% rhodamine-label), 1 mg/ml casein and 2 mM GTP. For dynamic microtubule experiments where seeds were double stabilized with taxol and GMPCPP (Figure 4-Supplement 1c, Figure 5e, f) the flow cell was washed with at least 10 cell volumes of 1 mg/ml casein (with the exception of data in Fig 5f where to provide additional protection against catastrophe only 5 cell volumes were used) and then incubated for 5 minutes before flow through of 6 flow cell volumes of assay buffer. Experiments which are compared (in the same graph or table) in this paper were done in parallel using the same ‘master mix’ assay buffer with at least three independent repeats, with the exception of data in Fig S3a and b where 2 repeats were performed for each condition. For photobleaching analysis, 0.25 nM Kif18b-GFP in BRB80 was introduced to the flow cell and allowed to non-specifically adsorb to the surface. After 3 minutes, BRB80 with oxygen scavenger mix was used to wash away non-adsorbed motors. The sample chamber was imaged using the same conditions as described for single molecule assays.

### TIRF Microscopy Imaging

Imaging was performed on a Zeiss Axio Observer Z1 TIRF microscope using a 100 × NA 1.46 objective and either a Photometrics Evolve Delta electron-multiplying charge-coupled device camera or a Photometrics Prime 95B sCMOS camera controlled by Zeiss Zen Blue software. For single molecule experiments in Figure 1 and Supplementary Figure 3 an Optosplit III beam-splitter in bypass mode inserted before the camera provided a further 2x magnification. Depolymerization assays were performed over 10 minutes at 1 fps or 0.5 fps for two colour imaging, this had no effect on depolymerization rates. Microtubule dynamics assays were performed over 15 minutes in either one, two or three colours at 0.3 fps. Two colour imaging of Kif18b-GFP and SNAP_546_-MCAK_1-177_ was performed over 5 minutes at 0.5 fps. MCAK single molecule imaging was performed in the GFP Channel only at 10 fps (stable microtubules) or 6 fps (dynamic microtubules).

### Image analysis

Kymographs were produced using ImageJ (National Institutes of Health), MCAK single molecule behaviour and catastrophe length and catastrophe time were manually measured from these kymographs. When analysing microtubule dynamics kymographs, we were unable to consistently detect microtubule growth events that occurred for less than 25 seconds, so these data were removed from our analysis. Line scan intensity measurements along microtubules were taken using a fixed threshold in the microtubule fluorescence channel (Rhodamine or Hilyte 647) to specify the microtubule ends. Intensity measurements for Kif18b motors were taken from kymographs and represent the background subtracted maximum intensity in the GFP channel along the trajectory of the motor. Graphpad Prism 9 was used for all statistical analysis.

### Statistics and reproducibility

Statistical analyses were performed using Prism 9 (GraphPad Software). For the calculation of the error on the median, we report the upper 95% confidence interval. No statistical method was used to predetermine sample size. No samples were excluded from the analyses. The investigators were not blinded to allocation during experiments and outcome assessment. All experiments were performed and quantified from at least three independent experiments (unless specified otherwise), and representative data are shown.

**Table:** Key Resources Table

Reagent type (species) resource	Designation	Source or reference	Identifiers	Additional information
strain, strain background *(Escherichia coli)*	BL21(DE 3) codon+	Agilent	Cat. #: 200131	Competent cells
strain, strain background *(Escherichia coli)*	X10 gold	Agilent	Cat. #: 200314	Competent cells
cell line *(Homo- sapiens)*	Hela	Sigma Aldrich	Cat. #: 93021013	
cell line (Sf9)	Sf9	Novagen	Cat. #: 71104-3	
Recombinant DNA reagent	mRuby3, Sf9 codon optimized	Geneart, Thermo Fisher	Sequence in this paper	
other	GTP	Sigma Aldrich	Cat. #: G8877-1G	nucleotide
other	GMPCPP	Jena Biosciences	Cat. #: NU405S	Nucleotide analogue
Software, algorithm	ImageJ	ImageJ (http://imagej.nih.gov/ij/)	RRID:SCR_0030 70	
Software, algorithm	GraphPad Prism	Graphpad	https://www.graphpad.com/scientific-software/prism/RRID:SCR_015807	
Recombinant protein	Rhodami ne-tubulin	Cytoskeleton, inc	Cat. #: TL590M-B	
Recombinant protein	Hil_yte647 -tubulin	Cytoskeleton, inc	Cat. #: TL670M	
other	SNAP-Surface Alexa Fluor647	NEB	Cat. #: S9136S	Chemical fluorophore
other	SNAP-Surface Alexa Fluor546	NEB	Cat. #: S9132S	Chemical fluorophore
other	Catalase from Bovine liver	Sigma-Aldrich	Cat# : C40	enzyme
other	Glucose oxidase from Aspergillus niger	Sigma-Aldrich	Cat# :G2133	enzyme
antibody	anti-MCAK (rabbit polyclonal)	Eurogenetec, purified in house	This study	IF(1:1000)
antibody	anti-EB1 (Mouse polyclonal)	BD biosciences	Cat#610535, RRID:AB 397892)	IF(1:400)

### Supplementary Material

Supplementary Materials
