# Supplementary material for: Potent microtubule depolymerizing activity of a mitotic Kif18b-MCAK-EB network
Source: J Cell Sci. Author manuscript; Available in PMC 2025 Jul 24. (PMC7617936; doi:10.1242/jcs.260144)

Supplementary Figure 1

a) Representative immunofluorescence images of control Hela cells and Kif18b-knockout HeLa cells stained for DNA, EB1 and Kif18b. Scalebar, 5 μm. b and c) Alphafold2 predicted models of monomeric MCAK and Kif18b drawn as cartoon models. b) Model of MCAK (PDB:AF-Q99661), the N terminus 1-190 is coloured magenta, with the EB binding motif SKIP and the residues phosphorylated by Aurora kinases drawn as stick and balls according to elements. The neck linker, motor and C terminus are represented in green. The amino acids in the far C terminus essential for intramolecular interactions between the C terminus and the motor (Talapatra et al, 2015, Zong et al, 2016) are shown in blue stick and ball mode. c) Model of Kif18b (PDB:AF-Q86Y91), the N terminus is at the start of the motor domain in green, a dimeric domain (McHugh et al, 2018) is painted blue and the tail containing the EB binding region is of low complexity and represented in magenta. d) Coomassie stained gel of purified proteins used in the experiments in this paper. e) Coomassie stained gel showing Kif18b-GFP-His bound to Ni^{2+} beads after incubation and washing with SNAP-MCAK$_{1\text{-}177}$ and SNAP-MCAK$_{178\text{-}725}$.

Supplementary Figure 2

a) Dependence of maximum fluorescence intensity of Kif18b-GFP at microtubule plus ends on the length of microtubule, 0.5 μm bins, mean and S.E. n=42, Kif18b-GFP intensity showed a positive correlation with microtubule length. P-value<0.0001. b) Quantification of the GFP fluorescence intensity along the microtubule in the presence of 50 nM $SNAP_{647}$-EB3 and 12.5 nM (red, n = 41) or 0 nM (pink, n = 44) of Kif18b-GFP (mean and S.E). c) Quantification of mRuby3 fluorescence intensity (mean and S.E) along the microtubule in the presence of 12.5 nM Kif18b-mRuby3 alone (dark blue, n = 64), with 12.5 nM MCAK-GFP (blue, n = 90), with 12.5 nM MCAK-GFP and 50 nM $SNAP_{647}$-EB3 (purple, n = 116). Kolmogorov-Smirnov test at 0.2 μm, Kif18b vs Kif18b + MCAK, ns (P=0.1279), Kif18b vs Kif18b + MCAK + EB3, **** P<0.0001. d) Quantification of GFP fluorescence intensity along the microtubule in the presence of 50 nM GFP-PRC1 and 25 nM (dark blue, n = 26) or 0 nM (green, n = 26) of Kif18b-mRuby3 (mean and S.D). e) Representative images of Kif18b-mRuby3 (blue), GFP-PRC1 (green) localization on taxol- and GMPCPP-stabilized microtubules (HiLyte647- tubulin, red). Scale Bar 20 μm.

Supplementary Figure 3

a) Fluorescence intensity measurements median and 95% C.I. Kif18b-GFP on microtubules for static and processive Kif18b (n= 65 and 97 respectively). Asterisks indicate Kolmogorov-Smirnov significance *, P=0.0287. b) Corresponding intensity profile for a motor in a. Horizontal red lines show the mean intensity for the respective sections of the profile. c) Example kymographs for 0.25 nM MCAK-GFP and 4.75 nM unlabeled MCAK showing the increase in diffusive behavior in the presence of Kif18b-mRuby3. Scale bars, 10 seconds (vertical) and 3 μm (horizontal). Highlighted section shows tracks where MCAK moves directionally towards one end of the microtubule. d) Mean squared displacement plotted against the time over which it was measured for 0.25 nM MCAK-GFP motors in the presence of 4.75 nM unlabeled MCAK and 0 nM (green, n = 246) or 10 nM Kif18b-mRuby3 (blue, n =

820), mean and S.E. Fitted with a linear curve from which the diffusion coefficient D can be measured ($2Dt = \langle x^2 \rangle$). e) Frequencies of microtubule residency time for MCAK-GFP motors on GMPCPP stable microtubules with 0 nM (green, n=247) and 10 nM (blue, n = 820) Kif18b-mRuby3, mean and S.E. are fitted with exponential curves to give mean residency times $t_{1/2}$.

Supplementary Figure 4

a) Measured growth rates of dynamic microtubule extensions in the absence and presence of $Kif18b_{591-852.}$ b) Microtubule catastrophe frequency and c) length of dynamic microtubule extensions in the absence and presence of 500 nM $Kif18b_{591-852}$. For each condition, n=188 and 175 respectively, from 2 independent repeats. d) Measured growth rates of dynamic microtubule extensions in the absence and presence of increasing concentration of $Kif18b_{591-852.}$ e) Microtubule catastrophe frequency and f) length of dynamic microtubule extensions in the presence of 12.5 nM MCAK and with and without 500 nM $Kif18b_{591-852}$. For each condition, n=188 and 175 respectively, from 2 independent repeats. Kolmogorov-Smirnov test, P-value, **** P<0.0001, ** P=0.0097 for catastrophe frequency (e) and * P=0.0454 for microtubule length (c). Median and 95% C.I are presented.

Supplementary Figure 5

a) Representative kymographs of dynamic microtubules with 12 µM rhodamine tubulin, and 25 nM MCAK-GFP with or without 150 nM $SNAP_{647}$-EB3. Scale 5 mins (vertical) and 5 µm (horizontal). Seeds are taxol and GMPCCP stabilized. b) Representative kymographs of dynamic microtubules in the presence of 12.5 nM Kif18b-GFP and 12.5 nM MCAK-GFP with or without 25nM SNAP-EB3 with 12 µM tubulin. Scale 5 mins (vertical) and 5 µm (horizontal). c) Example kymograph for 5nM MCAK-GFP in the presence of 12.5nM Kif18b-mRuby3 on dynamic microtubule lattice showing both diffusive and processive behaviours. Scale bars, 30 seconds (vertical) and 5 µm (horizontal). d) Velocities of processive tracks of with 5 nM MCAK-GFP and 12.5 nM Kif18b-mRuby alone and with 25 nM $SNAP_{647}$-EB3 (n =13 and n=23 respectively). T-test, P-value, ns P= 0.2133. e) Percentage of MCAK-GFP microtubule binding

events lasting over 10 frames (1.6 seconds) that show processive behaviour with 5 nM MCAK-GFP, 12.5 nM Kif18b-mRuby alone and with 25 nM $SNAP_{647}$-EB3 (n=286 and 251 respectively). Two tailed Binomial Test, P-value, ** P = 0.002.

Supplemental Tables 1

Tables related to figure 3

Figure 3d**.** Summary of depolymerization rates, median and 95% C.I. and n values for the data shown in Figure 3d.

Figure 3e. Summary of depolymerization rates, median and 95% C.I. and n values for the data shown in Figure 3e.

Figure 3f. Summary of landing rates, median and 95% C.I. and n values for the data shown in Figure 3f.

Tables related to figure 4

Figure 4b-c. Summary of catastrophe frequency and microtubule length (median and 95% C.I. and n values). A tubulin concentration of 12 µM was used

Figure 4e-f. Summary of catastrophe frequency and microtubule length. A tubulin concentration of 7 µM was used (median and 95% C.I. and n values).

Figure 4h-i**.** Summary of catastrophe frequency and microtubule length. A tubulin concentration of 7 µM was used. (median and 95% C.I. and n values).

Table related to figure 5

Summary of catastrophe frequency and microtubule length in the presence of MCAK, Kif18b and EB3. A tubulin concentration of 12 µM was used. (median and 95% C.I. and n values).

Supplemental Tables 2

Table 1

Summary of the constructs and cloning details used in this study.

Table 2

List of the primers used in the creation of the constructs used in this study.

Video 1. Kif18b moves MCAK and EB3 to the plus ends of growing microtubules
Dynamic microtubules in the presence of 25 nM MCAK-GFP (green), 25 nM Kif18b-mRuby3 (red), 100 nM $SNAP_{647}$-EB3 (blue) with 12 µM tubulin. Seeds were stabilized with taxol and GMPCPP.

Supplementary Figure 1

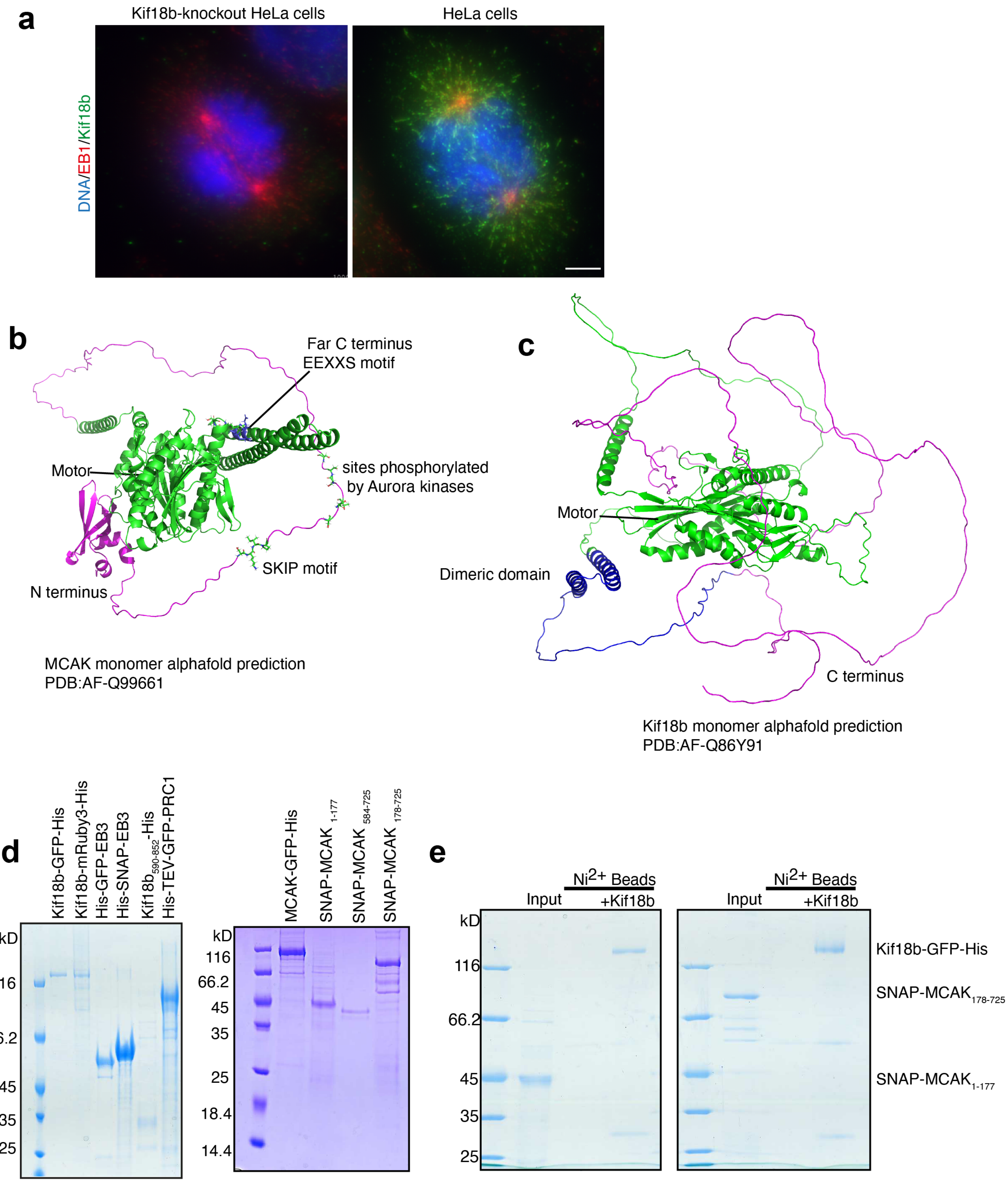

Supplementary Figure 2

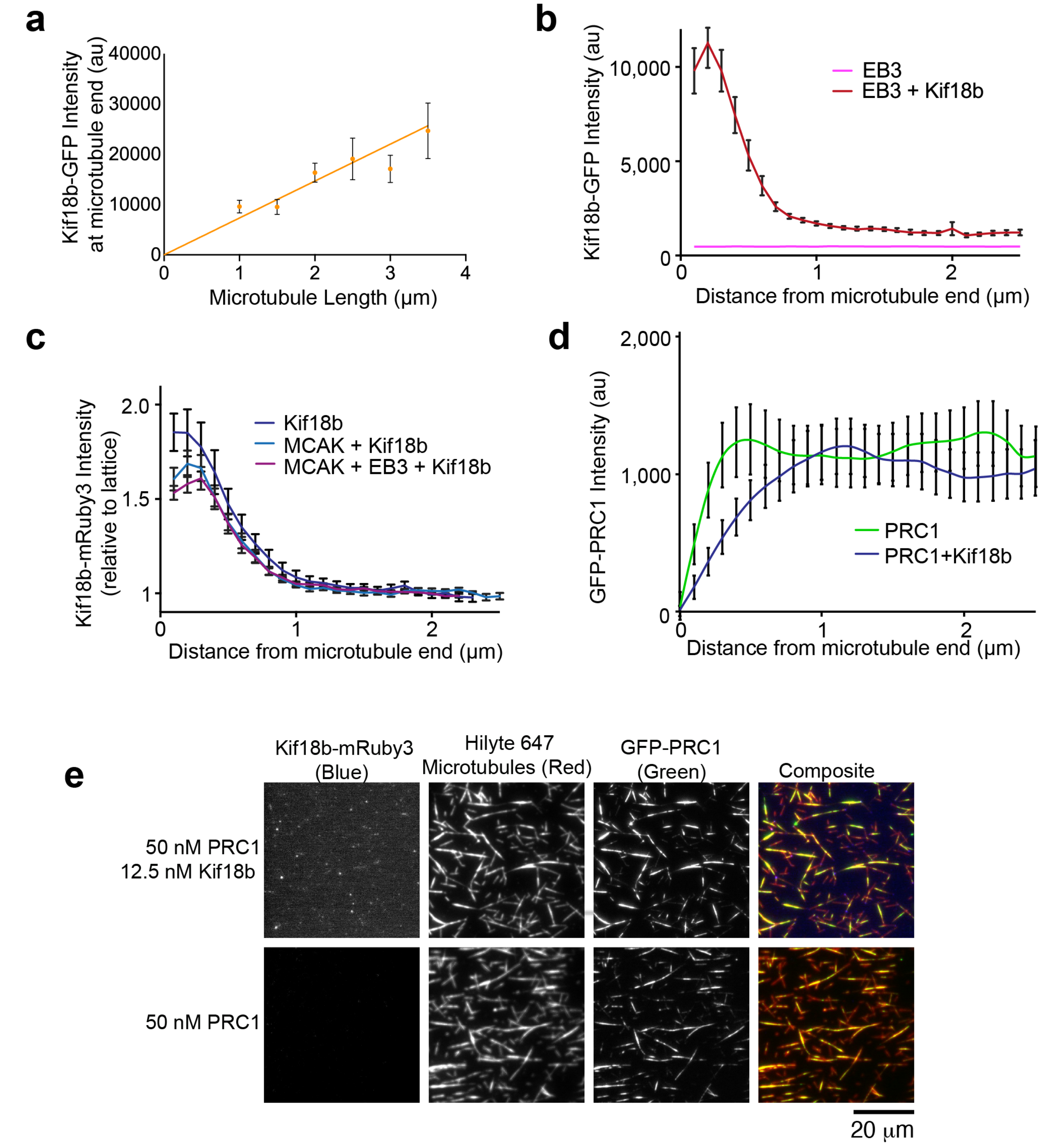

Supplementary Figure 3

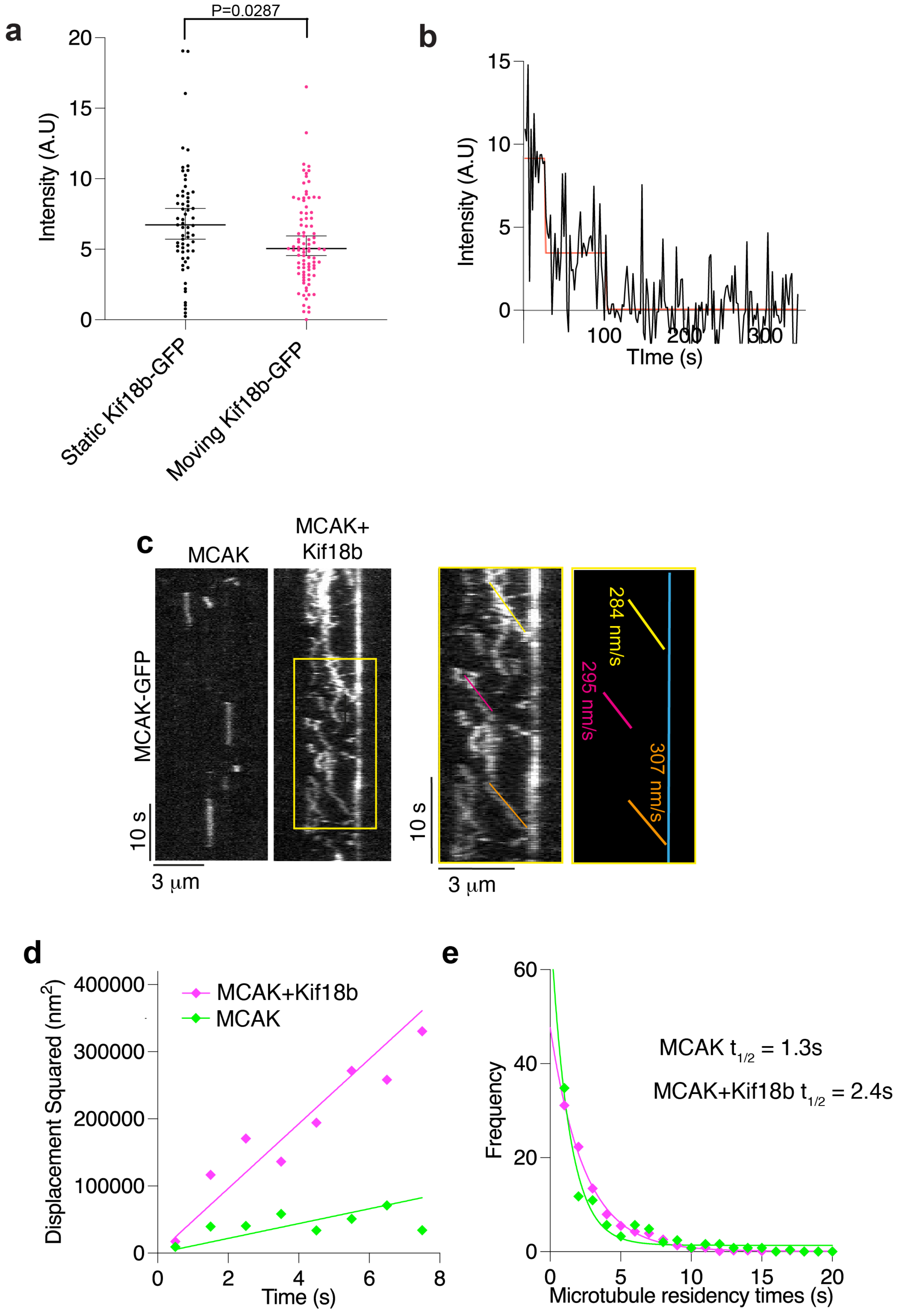

Supplementary figure 4

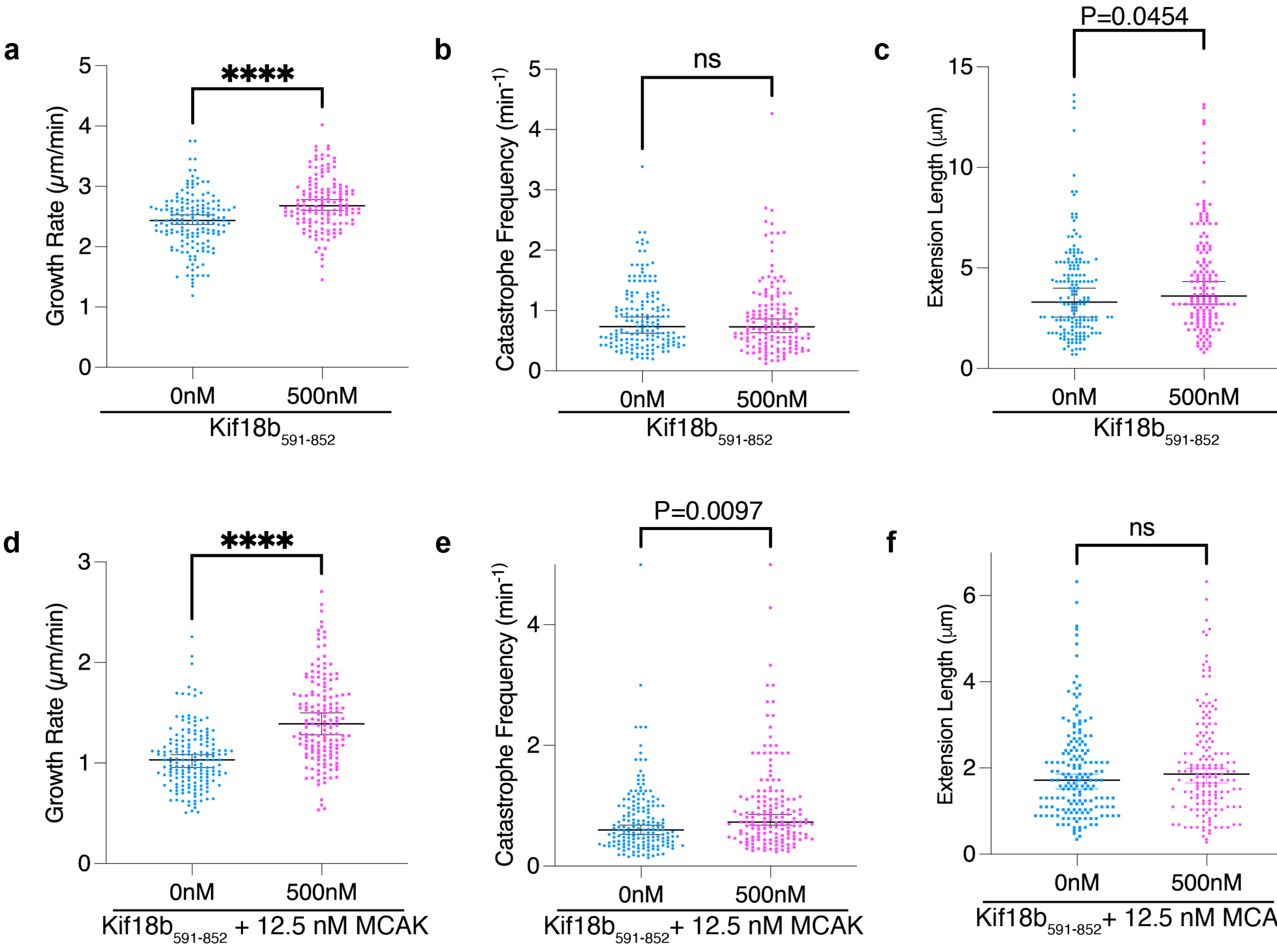

Supplementary Figure 5

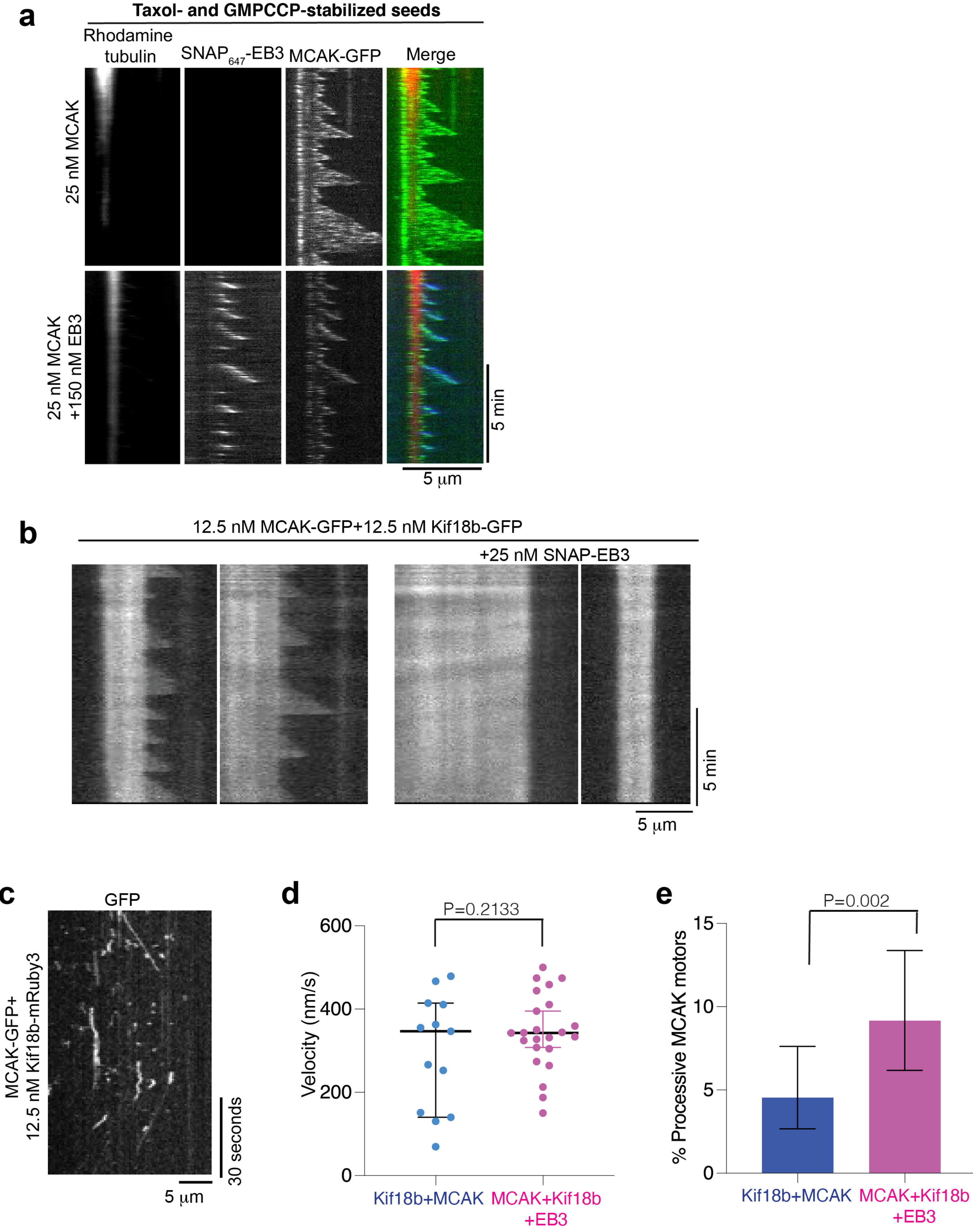

Supplement: Supplementary Materials [file EMS206576-supplement-Supplementary_Materials.pdf]
